# A Speech-Enabled Fixed-Phrase Translator for Emergency Settings: Crossover Study

**DOI:** 10.2196/13167

**Published:** 2019-05-07

**Authors:** Hervé Spechbach, Johanna Gerlach, Sanae Mazouri Karker, Nikos Tsourakis, Christophe Combescure, Pierrette Bouillon

**Affiliations:** 1 Geneva University Hospitals Genève Switzerland; 2 Faculté de traduction et d'interprétation Université de Genève Genève Switzerland

**Keywords:** anamnesis, emergencies, tools for translation and interpreting, fixed-phrase translator, speech modality

## Abstract

**Background:**

In the context of the current refugee crisis, emergency services often have to deal with patients who have no language in common with the staff. As interpreters are not always available, especially in emergency settings, medical personnel rely on alternative solutions such as machine translation, which raises reliability and data confidentiality issues, or medical fixed-phrase translators, which sometimes lack usability. A collaboration between Geneva University Hospitals and Geneva University led to the development of BabelDr, a new type of speech-enabled fixed-phrase translator. Similar to other fixed-phrase translators (such as Medibabble or UniversalDoctor), it relies on a predefined list of pretranslated sentences, but instead of searching for sentences in this list, doctors can freely ask questions.

**Objective:**

This study aimed to assess if a translation tool, such as BabelDr, can be used by doctors to perform diagnostic interviews under emergency conditions and to reach a correct diagnosis. In addition, we aimed to observe how doctors interact with the system using text and speech and to investigate if speech is a useful modality in this context.

**Methods:**

We conducted a crossover study in December 2017 at Geneva University Hospitals with 12 French-speaking doctors (6 doctors working at the outpatient emergency service and 6 general practitioners who also regularly work in this service). They were asked to use the BabelDr tool to diagnose two standardized Arabic-speaking patients (one male and one female). The patients received a priori list of symptoms for the condition they presented with and were instructed to provide a negative or noncommittal answer for all other symptoms during the diagnostic interview. The male patient was standardized for nephritic colic and the female, for cystitis. Doctors used BabelDr as the only means of communication with the patient and were asked to make their diagnosis at the end of the dialogue. The doctors also completed a satisfaction questionnaire.

**Results:**

All doctors were able to reach the correct diagnosis based on the information collected using BabelDr. They all agreed that the system helped them reach a conclusion, even if one-half felt constrained by the tool and some considered that they could not ask enough questions to reach a diagnosis. Overall, participants used more speech than text, thus confirming that speech is an important functionality in this type of tool. There was a negative association (*P*=.02) between the percentage of successful speech interactions (spoken sentences sent for translation) and the number of translated text items, showing that the doctors used more text when they had no success with speech.

**Conclusions:**

In emergency settings, when no interpreter is available, speech-enabled fixed-phrase translators can be a good alternative to reliably collect information from the patient.

## Introduction

### Background

In the context of the current refugee crisis, emergency services are increasingly confronted with patients who have no language in common with staff and may not share the same culture. For example, at Geneva University Hospitals (HUG), 52% of patients are foreigners and 10% speak no French at all. In 2017, the 10 languages for which interpretation services were the most solicited were Tigrinya, Tamil, Albanian, Farsi, Spanish, Somalian, Syrian, Dari, Portuguese, and Arabic (North Africa). Taken together, these languages represent 75% of the interpreting hours at HUG (Geneva University Hospitals, personal communication, 2017).

This language barrier situation is known to pose many safety and ethical problems: It is responsible for increased risks for patients [1] and is very expensive. For example, as reported by Rechel et al in 2003 [2], the United States Institute for Healthcare Advancement estimated that US $73 billion was wasted annually in the United States as a result of communication problems in health care, many of which originate from language differences. Both ethically and legally, hospitals have a duty to offer all patients the same quality of care, including the right to have a dialogue with health professionals.

Different solutions are available for use in emergency settings to address these language barriers, but they all have their drawbacks. Phone-based interpreter services, which are the most common solution, are generally considered adequate, but they are expensive (3 Swiss francs/minute with AOZ Medios, a national interpreting service mandated by the Swiss Federal Office of Public Health), not always available for some languages, and less satisfactory than face-to-face interaction with a physically present interpreter [3]. Asking patients’ relatives to translate speech is known to create substantial risks [1]. Machine translation, such as Google Translate, another low-cost solution more commonly used in emergency contexts, is also extremely problematic, as this type of tool has not been developed for medical use. Some recent studies have estimated that nearly 40% of sentences of medical speech translated by Google Translate are mistranslated [4,5]. However, such systems also pose ethical problems and are not currently compatible with the Swiss Data Protection Law. A plethora of specialized systems have also been developed for medical communication, both in the academic and industry settings (including fixed-phrase translation or machine translation systems [6]), but it is not always clear how they were built or evaluated and if they are extensible. As emphasized in the recent review by Dew et al [6], there is a lack of criteria for the development and evaluation of these systems, which impedes the adoption of these systems in emergency settings.

For these reasons, we have developed a new type of speech-enabled fixed-phrase translation tool for medical dialogue (BabelDr [7]), based on our previous experience in the field [8] in a collaborative venture between HUG and the University of Geneva Faculty of Translation and Interpreting. This tool is a compromise between speech-to-speech machine translation and fixed-phrase translation systems and directly addresses specific needs in emergency settings (ie, high accuracy, extensibility, portability to low-resource languages and domains, and data security). It was also designed as a way to collect doctor-patient dialogues and thereby improve our understanding of the criteria for the development of this type of system.

This study is the first step in this direction. It aims to determine whether this type of restricted translation tool can be used by doctors to perform a diagnostic interview and reach a correct diagnosis and to quantify if speech adds value to fixed-phrase translators. Although different evaluations of medical devices have been conducted [6], to the best of our knowledge, this is the first study that attempts to show the impact of “phraselators” on the diagnosis and to define a methodology to achieve this.

### The BabelDr App

The BabelDr app can be characterized as a “phraselator” [9,10]. Similar to well-known medical fixed-phrase translation apps such as Medibabble [11] or UniversalDoctor [12], the system relies on a set of predefined sentences (mostly yes/no medical questions or instructions) translated by human translators to ensure translation reliability. However, in contrast to traditional fixed-phrase translators, the doctor can also freely ask his/her question and the system will match the recognition result to the closest predefined sentence in the list. The app was designed from the beginning to meet the hospital’s needs. In particular, it is easy to extend it to new target languages and situations in order to follow demographic changes and allow its integration in different services. The content is described efficiently with rules (synchronized grammar [13]) that map multiple synonymous patterns (“variations”) to a sentence expressing the core meaning (“core sentence”). For example, “Do you have a fever?” “Is your temperature high?” and “Have you observed a high temperature?” will all be mapped to the core sentence “Do you have fever?” In addition, patterns with variables (eg, “Is it a QUALITATIVE pain?” “Do you have a QUALITATIVE pain?” etc, where “QUALITATIVE” is a variable that can take multiple values such as “severe” and “dull”) allow the description of content in a productive way. The system currently contains around 2500 patterns and 600 variables, linking more than one billion variations to approximately 25,000 core sentences. Translation follows the usual standards and is performed online with translation memory in two steps—translation of patterns followed by revision of complete sentences [14]. Target languages focus on the languages important for HUG (Spanish, Arabic, Swiss French sign language, Tigrinya, Farsi, Dari, and Albanian). To ensure data confidentiality, both speech recognition and translation are carried out on secure local servers and all interactions are saved locally.

For speech recognition and matching, the system combines rule-based and robust methods, derived from the rules. When the doctor speaks, the system first recognizes what is said using both a grammar-based version of “Nuance” and a specialized statistical version (Nuance Communications Inc, Burlington, MA). It then maps the recognition results to the closest core sentence using both rules and robust matching techniques borrowed from information retrieval, described in detail elsewhere [15]. This closest core sentence is then translated orally for the patient who will answer nonverbally. As it is not an exact translation of the doctor’s question, but a translation of the corresponding matched sentence, the core sentence is always echoed back to the doctor, so that he can verify what the system understood. The translation is thus only produced for the patient if the core sentence is approved by the doctor. Therefore, core sentences play a crucial role in the process by not only providing feedback to doctors concerning recognition accuracy, but also making the meaning of the sentence explicit for both translators and patients [16,17]. These core sentences were designed very carefully with doctors and translators, so that they are as accessible and explicit as possible in order to avoid communication problems. In addition to using core sentences for the verification of translations, users can also access them directly by browsing and searching via keywords. The associated translations can then be submitted without the need for further checking, similar to other phraselators [16].

**Figure 1:**
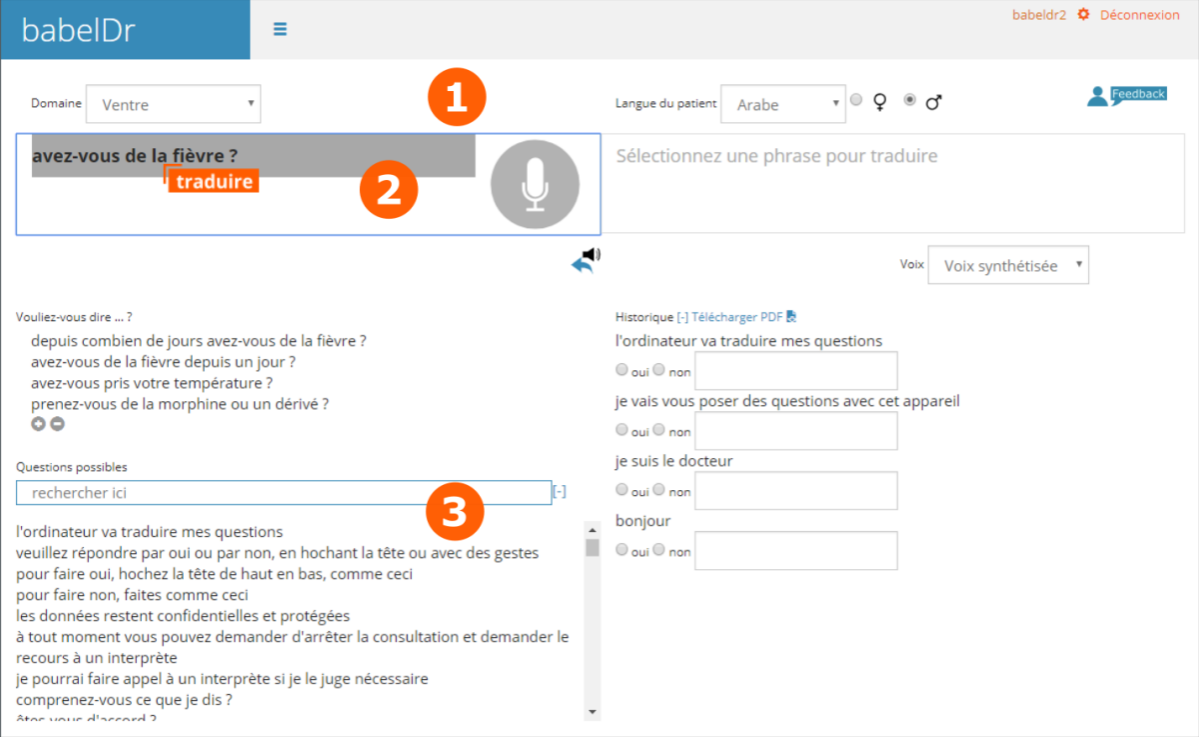
Screenshot of the BabelDr app.

Figure 1 illustrates the BabelDr interface and how an interaction is carried out. The doctor first selects the diagnostic domain based on the main patient complaint (headache, abdominal pain, dermatological problem, etc) and the language and gender of the patient (male or female). He/she can then speak a sentence (“speech interaction”). If the echoed core sentence corresponds to what the doctor wants to ask, he/she can click on it to produce the translation for the patient. In addition to speech input, doctors can search the list of core sentences using keywords (only with exact matching, as in traditional phraselators) and click on sentences to translate them for the patient (“text translated”).

**Figure 2:**
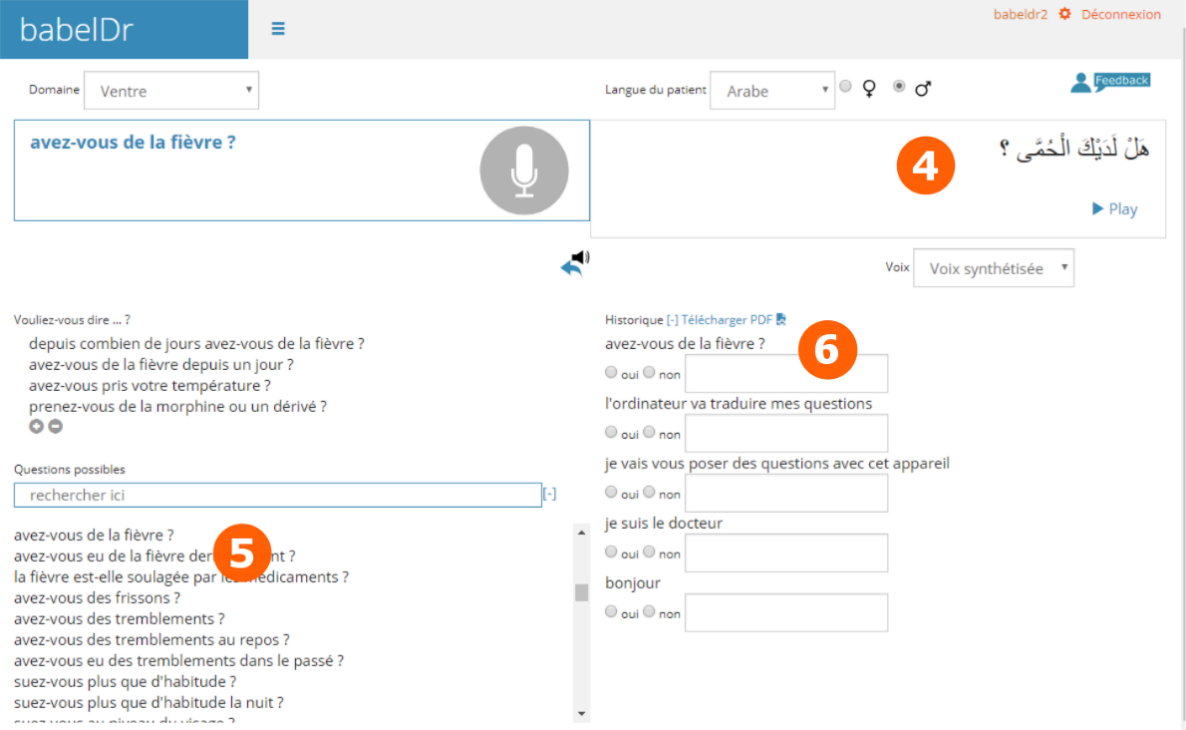
Example of the interface after the doctor translated “Avez-vous de la fièvre?” (Do you have fever?).

After translating a sentence to the patient (Figure 2), the translation is produced both in text and spoken form. The coverage list is automatically scrolled to the latest core sentence translated, giving quick access to related questions. The translated sentence is also added to a history list that can be downloaded as a PDF at the end of the dialogue.

## Methods

### Identifying the Research Questions

This study aims (1) to determine whether a restricted translation tool like BabelDr can be used by doctors to perform a diagnostic interview and reach a correct diagnosis and (2) to quantify how doctors use text versus speech interactions in order to investigate if speech adds value to fixed-phrase translators. Our hypotheses were that this type of tool would demonstrate good functional suitability (doctors can collect all the information necessary to reach a diagnosis in an efficient way) and usability (doctors will use more speech to interact than text, as speaking should allow them to communicate more naturally, like when working with interpreters).

### Design

The study was conducted at the HUG research laboratory in December 2017. In this crossover trial, 12 French-speaking doctors were asked to use BabelDr to diagnose two standardized Arabic-speaking patients (one male and one female) whose main complaint was lower back pain. The male patient was standardized for nephritic colic and the female patient, for cystitis. These two diagnoses are among the 10 most frequent at HUG (Geneva University Hospitals, personal communication, 2018). Each of the 12 doctors carried out a diagnostic interview with both patients, where half of the doctors began with the male patient and the other half began with the female patient.

Before the diagnostic interviews, doctors were informed about the main patient complaint (pain in the lower back). At the beginning of the session, they received a short introduction to BabelDr and tested a few interactions. It was strongly suggested that they use complete sentences and ask yes/no questions, so that the patients could answer nonverbally.

### Tool and Interface

Doctors only had access to the BabelDr tool. The diagnostic domain was set to “lower back pain” to match the patient complaint. In the context of this study, the other domains were not made available in order to simplify system usage. It was ascertained beforehand that all available questions potentially relevant to the patient complaint were included in this domain. The language pair was French to Arabic; the male or female patient was chosen depending on the case.

### Data Collection and Analysis

#### Diagnoses

During the sessions, the doctors wrote down the information they were able to collect based on the patient’s responses. At the end of each session, the doctors wrote down their diagnoses. These data allowed us to answer the first question on whether the system enables doctors to reach a correct diagnosis.

#### System Usage

All interactions with the system were logged. For each session, we collected audio recordings of each spoken interaction with the system as well as the corresponding recognition results. We also logged which recognition results or text examples the doctors chose to translate for the patients. Finally, the duration of each session was measured. These data were analyzed to provide a quantitative answer to our second research question, namely, whether speech interaction is useful in this type of tool.

#### User Satisfaction

At the end of each session, participants completed a satisfaction questionnaire that included a total of 23 questions. The questions were derived from the System Usability Scale questionnaire by Brooke [18] and adapted to the functionalities of BabelDr, especially the speech and core sentence mapping aspects. Questions covered usability and learnability aspects of the BabelDr system during the study (7 items), appropriateness of the system to confidently reach a diagnosis (6 items), the speech component of the system (3 items), and the user’s opinion regarding the usefulness of such a system in their daily medical practice (7 items). A 5-point Likert scale (“strongly disagree,” “disagree,” “neutral,” “agree,” and “strongly agree”) was used to rate agreement with question items. These data contribute to a qualitative answer to our second research question.

### Participants

#### Doctors

Study participants were 12 French-speaking doctors: 6 from the emergency service at HUG and 6 general practitioners who also regularly work in this service. All work in French, but three were not native speakers (#6, #11, #12). Only one doctor (#6) had previously used a former version of BabelDr in another study [5].

#### Standardized Patients

Of the two Arabic standardized patients, one was a man from Syria and one was a woman from Jordan. Both were refugees and recruited from among master’s degree students at the Faculty of Translation and Interpreting. They had a high level of literacy, but no specific medical knowledge. Neither of the patients spoke French. One week before the experiment, both patients received an a priori list of symptoms for the condition they were to present, expressed in layman’s terms. They were instructed to provide a negative or noncommittal answer to questions relating to other symptoms during the diagnostic interview.

All participants received remuneration for their participation in the study.

### Ethical Considerations

The institutional ethics committee approved the study protocol (Req-2017-00996). Participation in the study was voluntary, with written agreement obtained from all doctors and patients. All data were anonymous and stored on a secure University of Geneva server.

## Results

### Diagnoses

Doctors were able to reach a correct diagnosis in all 24 sessions based on the information collected using BabelDr. For the renal colic scenario, four doctors proposed multiple related diagnoses (Table 1). These results showed that BabelDr was suitable for the task and allowed doctors to collect information reliably.

Textbox 1 gives examples of the most frequently asked questions for each scenario. In total, more questions were translated for the renal colic scenario than for the cystitis one (170 vs 126 unique interactions, respectively), probably reflecting the fact that the first scenario was more complex due to a larger number of possible related diagnoses and thus required more different questions.

**Table 1:** Diagnoses made by the 12 doctors.

Doctor no.	Female patient (with cystitis)		Male patient (with renal colic)	
	Diagnosis	Other diagnoses	Diagnosis	Other diagnoses
1	Cystitis	No	Renal colic	Pyelonephritis
2	Cystitis	No	Renal colic	No
3	Cystitis	No	Renal colic	No
4	Cystitis	No	Renal colic	No
5	Cystitis	No	Renal colic	No
6	Cystitis	No	Renal colic	Lumbosciatica
7	Cystitis	No	Renal colic	No
8	Cystitis	No	Renal colic	No
9	Cystitis	No	Renal colic	Pyelonephritis, lumbosciatica
10	Cystitis	No	Renal colic	No
11	Cystitis	No	Renal colic	No
12	Cystitis	No	Renal colic	Pyelonephritis, lumbosciatica, appendicitis

**Most frequently translated core sentences for each scenario, sorted by frequency.**

**Female patient with cystitis:**

- *Pouvez-vous me montrer avec le doigt où est la douleur?* [Could you point with your finger to where it hurts?]
- *Avez-vous déjà eu ce type de douleur?* [Have you already had this type of pain?]
- *Bonjour* [Hello]
- *Je suis le docteur* [I’m the doctor]
- *Quand vous urinez, est-ce que ça brûle?* [Do you feel a burning sensation when you urinate?]
- *Avez-vous eu de la fièvre dernièrement?* [Have you had fever recently?]
- *Je vais m'occuper de vous aujourd'hui* [I will take care of you today]
- *Avez-vous mal au niveau des reins?* [Do you have pain in the kidney area?]
- *Je vais vous poser des questions avec cet appareil* [I will use this machine to ask you some questions]
- *Vos urines sont-elles rouges?* [Is your urine red?]
- *Êtes-vous d'accord?* [Do you agree?]
- *Il y a combien de semaines que vous avez eu vos dernières règles? * [How many weeks ago did you have your last period?]
- *Avez-vous été traité par antibiotique pour l'infection urinaire?* [Have you had antibiotic treatment for a urinary tract infection?]
- *Avez-vous eu une infection urinaire dernièrement?* [Have you recently had a urinary tract infection?]
- *Avez-vous des allergies connues?* [Do you have any known allergies?]

**Male patient with renal colic:**

- *Bonjour* [Hello]
- *Je suis le docteur* [I’m the doctor]
- *Vos urines sont-elles rouges* [Is your urine red?]
- *Avez-vous déjà eu ce type de douleur* [Have you had this kind of pain before?]
- *Pouvez-vous me montrer avec le doigt où est la douleur* [Could you point with your finger to where it hurts?]
- *Avez-vous eu de la fièvre dernièrement* [Have you had fever recently?]
- *Avez-vous mal au niveau des reins* [Do you have pain in the kidney area?]
- *La douleur aux reins irradie-t-elle vers un autre endroit* [Does the pain in the kidney area spread to any other place?]
- *Quand vous urinez, est-ce que ça brûle* [Do you feel a burning sensation when you urinate?]
- *Je vais vous poser des questions avec cet appareil* [I will use this machine to ask you some questions]
- *La douleur aux reins est-elle continue* [Is the pain in the kidney area continuous?]
- *Êtes-vous d'accord* [Do you agree?]
- *Je vais m'occuper de vous aujourd'hui* [I will take care of you today]
- *Depuis combien de jours avez-vous mal aux reins* [For how many days have you had pain in the kidney area?]
- *Avez-vous de la fièvre* [Do you have fever?]

### Analysis of Interactions

For each doctor, we measured the time to complete the dialogue, the number of speech interactions, the number of speech interactions resulting in a translation for the patient, and the number of text items directly translated from the list of sentences. Table 2 shows that both the median time and the median number of translated speech interactions were higher for the renal colic scenario (16 min for 26 interactions) than for the cystitis scenario (13 min for 19 interactions), confirming the fact that the renal colic scenario was more complex.

Table 2 shows that doctors translated both speech and text, but used more speech interactions, suggesting that speech was generally preferred to text. The median number of speech interactions per dialogue that led to translations was 28.5 for the cystitis scenario and 36 for the renal colic scenario, whereas the median numbers for text interactions were 4.5 and 10, respectively.

**Table 2:** Time and number of interactions for both scenarios.

Variable	Female patient with cystitis, median (range)	Male patient with renal colic, median (range)
Time to diagnosis (min:seconds)	13:37 (4:09-35:37)	16:37 (4:35-23:35)
Speech interactions (n)	28.5 (17-46)	36 (20-66)
Speech translated (n)	19.5 (8-23)	26.5 (13-51)
Text translated (n)	4.5 (0-36)	10 (0-23)

**Figure 3:**
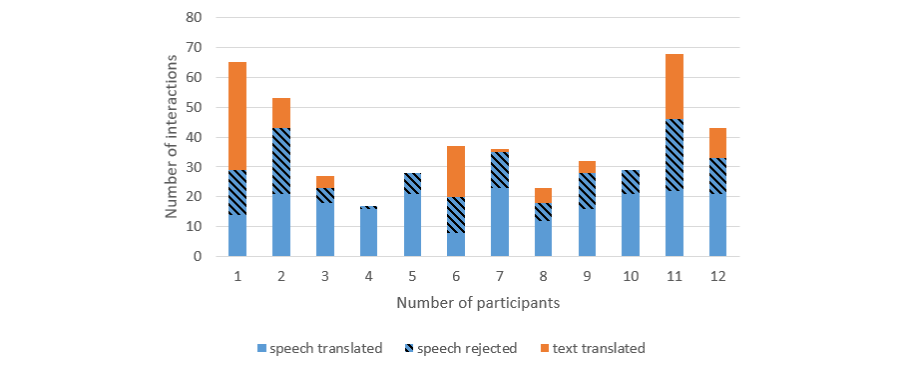
Interactions by participant for the scenario with the female patient.

**Figure 4:**
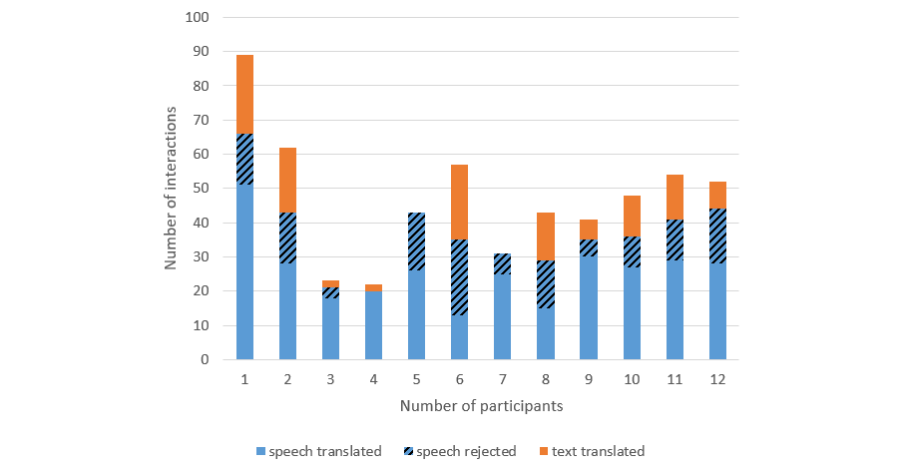
Interactions by participant for the scenario with the male patient.

Figures 3 and 4 present the interactions by participant and show that some used the text mode more often than others and that the number of speech sentences sent to translation differed from one participant to another. For different doctors, the proportions of recognition results leading to translations varied from 40% (8/20) to 94% (16/17) for the cystitis scenario and 37% (13/35) to 100% (20/20) for the renal colic scenario.

The association between the percentage of translated speech and the number of translated texts was investigated using a linear regression model. Since each medical practitioner assessed two patients, data were clustered. Therefore, a regression model with mixed effects was used: A random effect was set on the intercept to account for between-practitioner variability. In addition, a multivariable analysis was conducted to adjust for the session and the scenario.

**Figure 5:**
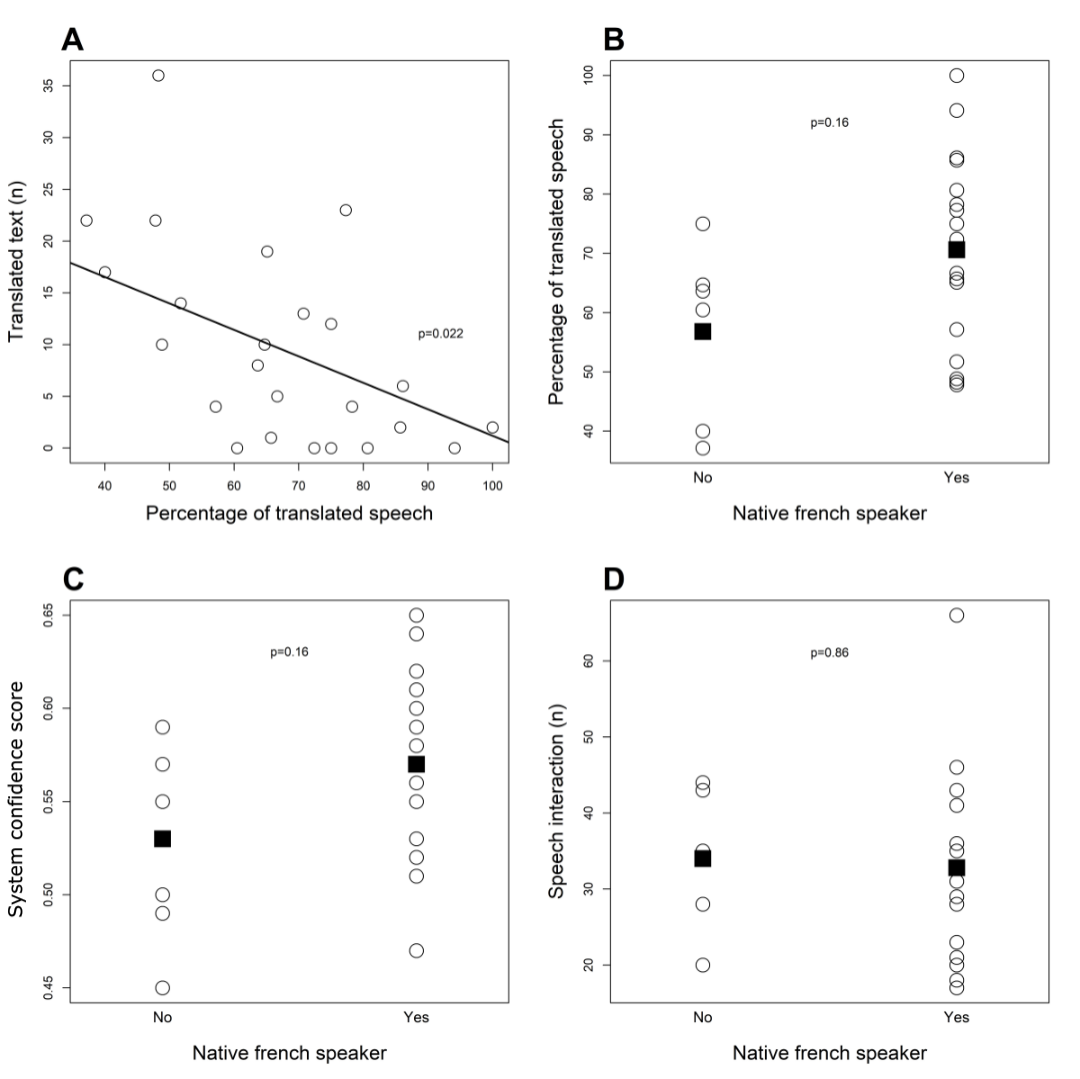
Association between the percentage of translated speech and the number of translated texts (A) and between French native speakers and the percentage of translated speech (B), system confidence score (C), and speech interaction (D). Circles represent each individual doctor's data; the black line represents the unadjusted regression line and black squares represent the mean values.

The percentage of translated speech was negatively associated with the number of translated texts (*P*=.02): When the percentage of translated speech increased by 10%, the number of translated texts decreased by 2.6 (95% CI 0.7-4.4). After adjustment for the session and scenario, the decrease in the number of translated texts was similar (2.4; 95% CI 0.7-4.2; *P*=.02). This association is illustrated in Figure 5A. These results show that users who are not well recognized tend to use the text interface more often, thereby confirming the usefulness of including both modalities in such a tool.

The percentage of translated speech was higher in the second session than in the first session (difference=4.3%; 95% CI 1.1-7.4; *P*=.03). One possible interpretation may be that users familiarized themselves with system coverage in the first session and therefore used more coverage utterances in the second session, leading to better recognition of results and thus more translations.

Analyses by scenario showed that the proportion of translated speech was lower in the renal colic scenario than in the cystitis scenario (difference=4.3%; 95% CI –7.6 to –1.1; *P*=.03). This may be due to different factors such as concepts not covered by the system at the time of the study or errors in speech recognition or mapping to the core sentences (eg, cases where a sentence is badly recognized and therefore mapped to a different sentence). In some cases, the core sentence could also be too general or specific or considered inappropriate in the context. Table 3 presents some examples of these cases.

**Table 3:** Examples of transcriptions and mapped core sentences.

Speech utterances		Core sentences
**Sent to translation**		
	*Est-ce que vous avez fait du sport* (Did you practice any sports?)	*Faites-vous de l’exercice* physique (Do you exercise?)
	*Est-ce qu’il y a du sang dans les* urines (Is there any blood in your urine?)	*Les urines sont-elles rouges* (Is your urine red?)
	*Est-ce que vous avez beaucoup transpiré* (Did you sweat a lot?)	*Suez-vous plus que d’habitude* (Do you sweat more than usual?)
	*Est-ce que c’est aujourd’hui* (Is it today?)	*Avez-vous mal depuis aujourd’hui* (Do you have the pain since today?)
	*Est-ce que vous avez des pertes vaginales particulières* (Have you observed any particular vaginal discharges?)	*Avez-vous des pertes blanches en dehors des règles* (Have you observed any white discharges outside normal menstruation?)
**Not sent for translation by at least one doctor**		
	*Avez-vous bu* (Have you had anything to drink?)	*Avez-vous bu de* l’alcool (Have you consumed any alcohol?)
	*Est-ce que vous pourriez être enceinte* (Could you be pregnant?)	*Êtes-vous enceinte* (Are you pregnant?)
	*Avez-vous du prurit* (Do you have pruritus?)	*Avez-vous des* démangeaisons (Do you have itchiness?)
	*La douleur est-elle constante* (Is the pain constant?)	*La douleur est-elle continue* (Is the pain continuous?)

Associations between French native speakers and the percentage of translated speech, system confidence, and speech interaction were also investigated using a linear regression model with fixed effects. No association was found between French native speakers and the percentage of translated speech (*P*=.16), system confidence (*P*=.16), and speech interaction (*P*=.86). Figure 5B-D illustrates these numbers. These results suggest that system performance is not significantly impaired by different accents.

### User Satisfaction

**Figure 6:**
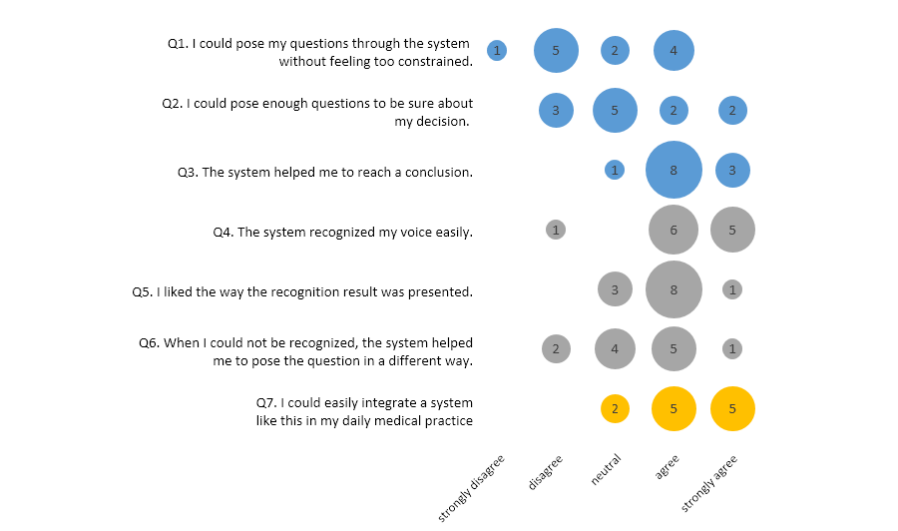
Results of the satisfaction questionnaire completed after the dialogue with the female patient. The numbers in circles represent the number of doctors.

**Figure 7:**
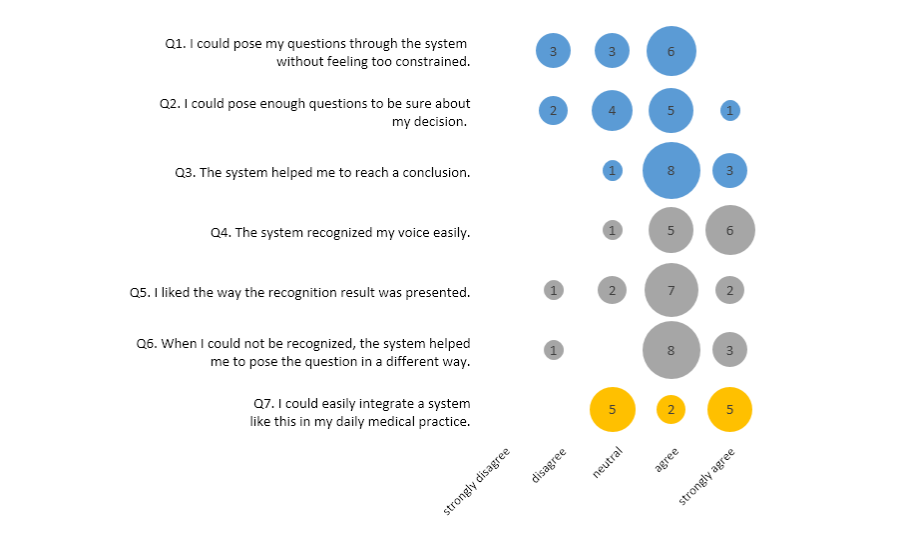
Results of the satisfaction questionnaire completed after the dialogue with the male patient. The numbers in circles represent the number of doctors.

Figures 6 and 7 show the results for seven questions related to the usefulness of the system for the diagnostic task and speech recognition included in the satisfaction questionnaires completed by the doctors after each dialogue (24 completed questionnaires). Overall, the doctors were satisfied with the speech interaction function and the usefulness of the system in the test context (19 negative, 54 neutral, and 116 positive judgments). All doctors considered that the system helped them reach a conclusion (Q3). They also liked the way the recognition result was presented (only one participant disagreed), which showed that they found the translation to the core sentence useful. All doctors thought that the system recognized their voice easily (Q4), and most believed that the system helped them to pose the question in a different way when the question could not be recognized (Q6: only 3 “disagree”). The most frequent criticism was that some doctors felt constrained by the tool (n=9/24) and were unable to ask all the questions they wanted to (5/24). In this respect, we observed differences between the two scenarios, suggesting that this issue is related to the system coverage or mapping of sentences to core sentences. Finally, all doctors believed that they could integrate such a system in their daily practice (Q7: no “disagree” or “strongly disagree”).

## Discussion

### Principal Results

All participants were able to pose their questions to the patients and reach the correct diagnosis based on the information collected using BabelDr. However, although they believed that the system helped them to reach a conclusion, some felt constrained by the tool, as they could not ask enough questions to reach a diagnosis. Speech was the preferred modality, even if all doctors translated items from the text list, thus showing that both modalities are useful. The use of text was statistically influenced by the percentage of successful speech interactions and by the session (first use vs second use). Therefore, speech seems to help in using the system, as participants can express themselves freely and see the most related core sentences.

### Comparison With Previous Research

Other studies have analyzed user satisfaction (of both patients and medical staff) [19,20] or the quality of translation with translation systems [4]. However, to our knowledge, this study is the first to measure the impact of the medium on diagnosis. This study confirmed the results of two previous evaluations of BabelDr. A comparison with a traditional fixed-phrase translator (Medibabble) in artificial settings (doctors had to find answers to specific questions) [21] showed that speech improves both usability (reduces time and number of clicks required to ask a question) and satisfaction. Another study [5,22] compared an earlier version of BabelDr with Google Translate at the level of diagnosis, satisfaction, and translation quality in a setting similar to this study. The main result was that BabelDr produced a better translation quality, improved precision (odds ratio: 0.04, 95% CI 0.02-0.12; *P*<.001 in favor of BabelDr) and fluidity (odds ratio: 0.04, 95% CI 0.02-0.10; *P*<.001 in favor of BabelDr) and led to more correct diagnoses than Google Translate.

### Limitations

A preliminary version of the tool was used in the study. The system coverage, that is, the questions available to the doctors, is being continually improved based on the collected data. It is possible that the perception of constraint reported by the users was at least partially caused by insufficient coverage for the scenarios selected for this study, rather than by the system itself.

For the cystitis scenario, doctors would have benefited to have been able to change to another domain (abdominal pain), which was not accessible for this study. In addition, the doctors were informed beforehand of the patient’s chief complaint. This matches the usual practice at HUG where this information is collected from patients during admission, but another study without prior information would ascertain whether the subdivision into domains, as done in BabelDr, meets the doctor’s requirements.

The two standardized patients had a higher education level and no difficulty understanding the Arabic translations provided by the system. In the case of less literate patients, misunderstandings might cause incorrect patient responses and thus lead to incorrect diagnoses. Although the BabelDr translations are aimed at simplicity, a study of the translation quality and accessibility is currently in progress to ascertain whether the translations are suited to patients of different ages, education levels, and cultural and geographic origins.

Due to the rehearsed nature of the patient narratives, based on the given lists of symptoms rather than the potentially vague or contradicting observations by a real patient, it can be argued that the system performance in terms of diagnostic success would be lower with real patients. However, we suspect that the system’s restriction to yes/no questions might actually improve clarity by enforcing precise questions and unambiguous patient responses.

During this experiment, we observed very few user errors, such as doctors forgetting to shut off the microphone or using questions that could not be answered nonverbally. Anecdotally, we have observed more such errors in real-use cases with real patients. However, it is possible that in the artificial setting of this study, doctors were more attentive to the system than when using it with a real patient, where the focus would be more on the patient, and thus, the proportion of successful interactions might be lower.

The number of dialogues per doctor (n=2) in this study was insufficient to measure a quantifiable learning effect, but a study is currently in progress at HUG, where BabelDr is used in real settings and the collected data will allow us to study its learnability.

### Future Research

Our results show that speech and text interaction are complementary in a tool such as BabelDr. Future developments of the system include an improved text-search module providing more flexibility than the current keyword search.

Development of a bidirectional version of the system is ongoing. In this new version, patients will have an interface where they are presented with a range of responses (eg, numeric values, colors, and pictograms). This will allow us to extend the questions available to the doctors by including open questions and will possibly reduce doctors’ feelings of being constrained by the system.

### Conclusions

This study showed that a phraselator can be an alternative to machine translation and traditional fixed-phrase translators to reliably collect information from the patient in situations where no interpreter is available. Although doctors felt constrained by the system, they were able to confidently reach a diagnosis, and all believed they could use this type of system in everyday medical practice. The relevance of task-based evaluation to assess the usefulness and usability of translation tools for the diagnosis task was also demonstrated and confirms the importance of reliability in this type of oral context. Doctors clearly appreciated the way in which speech recognition results were presented in the form of a back translation to French, which provided the exact meaning of the translation produced for the patient. Future studies with BabelDr have to confirm these conclusions in real-life settings and investigate the proportion of cases that can be reliably diagnosed with such a tool.

## Abbreviations

HUG: Geneva University Hospitals
